# Prevalence of Good Condom Usage and Its Association with Condom Use Self-Efficacy among Youth Attending HIV/STDs Clinics in Primary-Care Settings in Malaysia

**DOI:** 10.3390/ijerph191912179

**Published:** 2022-09-26

**Authors:** Norbainun Che Hamid, Khasnur Abd Malek, Nafiza Mat-Nasir, Mariam Mohamad, Nik Munirah Nasir

**Affiliations:** 1 Department of Primary Care Medicine, Faculty of Medicine, Sungai Buloh Campus, Universiti Teknologi MARA (UiTM), Sungai Buloh 47000, Malaysia; 2 Department of Public Health Medicine, Faculty of Medicine, Sungai Buloh Campus, Universiti Teknologi MARA (UiTM), Sungai Buloh 47000, Malaysia

**Keywords:** condom use self-efficacy, youth, primary care, Malaysia, HIV

## Abstract

The low prevalence of condom usage among youth with Human Immunodeficiency Virus/Sexually Transmitted Diseases (HIV/STDs) is a concern. Condom use self-efficacy has been identified as a significant predictor of condom usage. This cross-sectional study examines the prevalence of good condom usage and its association with condom use self-efficacy among Malaysian urban youth, aged between 18 to 24 years old attending HIV/STDs clinics in primary-care settings, Selangor. Utilising the Harmonised Malay version of Condom Use Self-Efficacy Scale (CUSES M-H) questionnaire, the data from 218 responders were analysed using univariate and multiple logistic regression. The prevalence of good condom usage was 61% (95% CI: 54%, 68%). The average mean score of condom use self-efficacy was 3.07. Condom use self-efficacy was divided into four subscales of mechanics, perceived barriers, assertiveness and intoxicants. The assertiveness subscale had the highest average mean score of 3.42, while the intoxicant subscale score had the lowest average mean score of 2.24. Good condom usage was significantly associated with condom use during first sexual intercourse (aOR = 5.81, 95% CI: 1.97, 17.14), duration diagnosis of HIV/STDs of more than 12 months (aOR = 6.40, 95% CI: 2.30, 17.86) and the high assertiveness subscale score (aOR = 1.19, 95% CI: 1.03, 1.36). A behavioural change campaign that targets high-risk youth in primary care settings could promote condom use self-efficacy particularly assertiveness to increase condom usage among the youth.

## 1. Introduction

Youth is known as a period of transition between childhood to adulthood that makes young people vulnerable to high-risk behaviour, particularly sexual behaviour [1]. In the local context, according to the National Health and Morbidity Survey Malaysia (NHMS) 2017, 7.3% of school-aged children had engaged in sex, with 31.9% having their first sexual intercourse before the age of 14 and 16.6% having two or more sexual partners [2]. Every year, an estimated 333 million new instances of curable, sexually transmitted disease (STDs) are reported worldwide, with the highest rate among people aged 20 to 24 [3]. Adolescents and young people are becoming a larger proportion of human immunodeficiency virus (HIV) positive people around the world with 400,000 young individuals aged 10 to 24 newly infected with HIV in 2020 alone [4]. In Malaysia, the prevalence of HIV among Malaysia’s young population, aged 13 to 29, has been on the rise since 2019 [5]. Meanwhile, similar trends were observed in STDs where the age-specific rates for adolescents aged 13 to 19 years for gonorrhea and syphilis were 8.8 per 100,000 people and 1.3 per 100,000 people, respectively, [6]. Over the last decade, the HIV epidemic in Malaysia has shifted from primarily People Who Inject Drugs (PWID) to sexual transmission, with sexual activity being the primary route of HIV infection among young people, particularly among young homosexual and bisexual men [7].

Although the barrier method is not 100% effective, using a condom properly and consistently can reduce the risk of transmission of HIV and some other STDs [8]. However, despite the increasing trend of HIV and STDs among youth, the low prevalence of condom usage among them is a concern. Earlier studies discovered a low level of condom usage among youth across the South East Asian region [9,10] as well as in developed countries [11,12]. Meanwhile, NHMS 2017 reported that 12.7% of sexually active school-aged students in Malaysia used condoms during their last sexual intercourse, which decreased from 32.2% compared to the previous survey in 2012 [2]. Available data in Malaysia revealed that 49% of sexually active students are unaware that condoms can reduce the risk of HIV and STDs transmission [13].

Several studies have been undertaken to determine the fundamental mechanism of condom non-use among youth. The factors discovered include reduced sexual satisfaction, difficulty using, feeling shy to buy condoms, inability to convince a partner to use condoms [14], cost of condoms, aversion to the condom, consumption of alcohol or use of drugs before sexual intercourse [15,16], lack of knowledge and skills in a sexual relationship [17], social stigma, moral and religious factors [15]. These factors have been identified as important components of self-efficacy of condom use [18].

Self-efficacy refers to people’s judgement of their ability to plan and execute essential actions to achieve specific outcomes [19]. Self-efficacy is a concept derived from social cognitive theory (SCT), where it has gained increasing recognition as a predictor of the changes and maintenance of health behaviour [20]. According to SCT, knowledge and skills are necessary but not sufficient for self-protective behaviour. This occurs because knowledge and skills have no direct impact on behaviour, but it is mediated by a cognitive appraisal process that results in a judgement of one’s ability to manage a difficult situation [21]. This self-efficacy concept in SCT has proven to be useful in understanding condom use among youth [22]. The belief that one is both capable of and likely to use condoms in sexual activities is known as condom use self-efficacy. The higher level of condom use self-efficacy, the more likely that an individual will consistently use condom [23].

Condom use self-efficacy has been identified in several studies as an important determinant of condom usage among the youth population. A study among university students found that self-efficacy components such as appropriation, assertiveness, pleasure, and intoxication can be used to predict condom use and condom use intentions among youth [24,25]. Another study demonstrated that condom use self-efficacy measures which include technical skills, image confidence, emotion control, purchase, assertiveness, and sexual control accurately predicted condom usage among adolescents [26]. Apart from being a significant predictor of condom usage, several studies also reported a significant association between condom use self-efficacy and condom usage [27,28,29].

HIV/STDs prevention programs in Malaysia have made great progress since the first reported case of HIV in 1986. The initial focus was on treatment rather than prevention until the HIV screening programme was initiated among key population in 1990. In parallel with the National Strategic Plan for Ending AIDS, several strategies have been implemented, which include the community-based and primary health care linked to hospital-based care. The dedicated HIV/STDs clinic setup in the primary care setting started in 2015 [5,30]. Within the Selangor state, to date there are 36 clinics offering HIV/STDs clinic service [5].

There are limited published data being conducted in Malaysia to evaluate the relationship between condom use self-efficacy level and condom usage, particularly among youth who have been identified as at risk. Thus, this research aims to address the gap by exploring the association between self-efficacy measures, Condom Use Self-Efficacy Scale Malay Version (CUSES-M) [31] and condom usage among the youth population that attends HIV/STDs clinics in Malaysia. Findings from this study may provide helpful information in designing focused intervention strategies targeted at condom use self-efficacy to improve the management and prevention of HIV and STDs.

## 2. Materials and Methods

### 2.1. Settings, Study Design and Sampling

This cross-sectional study included patients attending HIV/STDs primary-care clinics located in central districts of Selangor state. A total of 14 clinics are providing HIV/STDs clinic services in the districts. Six clinics with onsite specialised HIV/STDs services recording high patient loads were purposively selected. The selected HIV/STDs clinic is run by the Family Medicine Specialist. Patients attending the clinic are either by walk-ins, referred from the hospitals or non-governmental organisations (NGOs). The clinic collaborates closely with NGOs through community-outreach programs and community-based testing. Approximately 1300 patients attended these six HIV/STDs clinics annually, of which, around 30% are youth. Using convenient sampling, patients attending HIV/STDs clinics were invited to participate in this study between June 2021 and June 2022. Convenience sampling was selected due to time constraints and the limited number of patients who could participate in the study because of the COVID-19 situation during the study. However, in order to minimize sampling bias, we included only youth and excluded adults, ensuring that every youth patient listed on the HIV/STDs clinic registry during the data collection days was approached for participation.

### 2.2. Study Participants and Recruitment

The study included participants between the ages of 18 to 24 years old, able to read, speak and understand the Malay language. We excluded participants who had reported psychiatric illnesses that may impair their ability to answer the questionnaire. The psychiatric illness that was excluded referred to participants who were mentally unstable and had ongoing active disease, for example, acute psychosis or major depressive disorder in depressed moods, while participants who were mentally stable, for example, treated for major depressive disorder were included in the study. Trained on-site staff nurses working in a HIV/STDs clinic or representatives from non-governmental organizations (NGOs) that were attached to the HIV/STDs clinic approached all participants during the data collection duration and invited those who fulfilled the eligibility criteria to participate in the study. The participants were informed of the study’s objective and procedures. Consenting participants were given a patient information sheet. Following this, the participants completed a self-administered questionnaire that consisted of five sections: Section A, B, C, D and E. Section A targets the sociodemographic, i.e., the participants’ gender, date of birth, age, ethnic, marital status, religion, education level, occupation status, type of clinic visit and follow-up length; Section B targets the sexual or personal factors, i.e., the participants’ sexual activity in the past three months, sexual orientation, sexual practices, number of sexual partners, age of first sexual intercourse, condom use during their first sexual intercourse and awareness about their partner HIV’s status; Section C targets the clinical factors, i.e., the presence of current HIV or other sexually transmitted diseases (STDs), the duration of diagnosis and current treatment if they have current HIV or STDs, past history of STDs, alcohol consumption, illicit drug consumption and smoking status; Section D targets condom usage, i.e., condom use in the last sexual intercourse and whether they plan to use a condom in future sexual intercourse, and; Section E targets Harmonised Malay version of CUSES (CUSES M-H) i.e., condom use self-efficacy covering four subscales; mechanic (ten questions), perceived barriers (seven questions), assertiveness (seven questions) and intoxicants (three questions). On average, the participants took 10 to 15 min to complete the questionnaire. Measures were taken to maintain privacy during the conduct of the study. The participants were provided with a secluded but convenient space for them to complete the questionnaire and a sealed box for them to return the questionnaire anonymously. This process is to promote confidentiality and anonymity of the data. A research assistant was available on-site to attend to any query immediately.

To ensure the data integrity, while converting data from the questionnaire to digital format, we ensured that only researchers had access to the data during data collection and data transfer. The hardcopies of the questionnaire were stored and packed securely in secured and locked cabinet while the participants were made anonymous in SPSS analysis. In addition to that, about 80% of the data transferred would be randomly audited or double checked to ensure validity of the data, and we would ensure that the data were similar for both formats.

### 2.3. Study Instrument: Measurements of CUSES M-H

The Harmonised Malay version of CUSES (CUSES M-H) was used to measure the ability of the study participants to use condoms (self-efficacy of condom use) [31]. CUSES M-H has been validated among the Malaysian population; thus, it has good enough reliability to be used as a research tool. CUSES M-H has underwent content validation (I-CVI > 1.00), forward and backward translation, face validation and field testing resulted in 27 items in the questionnaire. The study conducted among adult HIV/STDs patients in a primary care clinic in Malaysia utilising CUSES M-H reported good internal consistency, with an overall Cronbach’ alpha coefficient of 0.878. The individual subscales of CUSES M-H have acceptable to good Cronbach alpha: 0.884 (mechanics), 0.848 (perceived barriers), 0.877 (assertiveness) and 0.686 (intoxicants). The test-retest reliability analysis showed moderate to excellent reliability with intraclass correlation coefficients (ICC) of >0.4 [31].

The psychometric properties of CUSES M-H measure condom use self-efficacy through four subscales, which are mechanics, perceived barriers, assertiveness, and intoxicants. CUSES M-H consists of 27-items. The mechanic’s subscale (10 items) indicated the participants’ self-efficacy in obtaining, using, and disposing of themselves or their partner’s condoms without reducing sexual pleasure. An example is in item 1, “I feel confident in my ability to put a condom on myself or my partner”. The perceived barriers subscale (7 items) reflected the physiological response aspect of self-efficacy that is triggered by perceived fear, low self-esteem, and fear of disapproval of partner regarding condom usage. An example is in item 7, “I would feel embarrassed to put a condom on myself or my partner”. The assertiveness subscale (7 items) measures assertiveness self-efficacy by having the ability and skills to negotiate condom usage with their partners. An example is in item 3, “I feel confident in my ability to discuss condom usage with any partner I might have”. The final subscale is intoxicant (3 items), which implies the participants’ ability to use a condom while under the influence of alcohol or drugs. An example is in item 24, 25 and 28; “I feel confident that I would remember to use a condom even after I have been drinking”, “I feel confident that I would remember to use a condom even if I were high” and “I feel confident I could stop to put a condom on myself or my partner even in the heat of passion”. Even though the intoxicant subscale has the least number of questions, the questions have been proven to measure intoxicant subscales accurately based on previous studies [18,25]. The CUSES M-H is graded on a 5-point Likert scale that ranges from 0 (strongly disagree) to 4 (strongly agree). After reversing for seven negatively worded items, the scores are summed to yield a total score ranging from 0 to 108, where higher scores indicate greater condom self-efficacy.

### 2.4. Operational Definition

The dependent variable of this study is good condom usage. Good condom usage in this study is defined by youth who reported ‘yes’ to both of these questions; “Did you use a condom in your last sexual intercourse?” and “Would you use a condom in future sexual activity?”.

### 2.5. Sample Size Calculation

The sample size was calculated based on the study by Ajayi et al.; the proportion of youth with poor condom use self-efficacy is 34% and the adjusted odds ratio of high condom self-efficacy score towards condom usage is 2.50 [32]. Using the OpenEpi Version 3.01 for “Sample Size: X-Sectional, Cohort, and Randomized Clinical Trials” with significant level of 0.05, confidence interval of 95% (2-sided) and the power of 80%, the minimum calculated sample size is 180.

### 2.6. Statistical Analyses

Data were managed and analysed using the IBM^®^ Statistical Package for Social Sciences (SPSS) version 27 program [33]. Categorical data were presented in frequency (n) and percentage (%), while mean and standard deviations (SD) were used to describe continuous data. Inferential analyses were used to determine the factors associated with good condom usage. Univariate analyses were conducted by using simple logistic regression and Chi-squared test to estimate the crude odds ratio (OR) of the factors associated with good condom usage. Variable with *p*-value < 0.05 was then further analysed using multiple logistic regression (MLogR) model using the backward method to adjust for the confounders. The factors associated with good condom usage were expressed as adjusted OR. Statistical significance was taken at *p*-value < 0.05. Model fitness was checked using the Hosmer–Lemeshow goodness-of-fit test. Interactions, multicollinearity and assumptions were also checked. Statistical significance was taken at a *p*-value of < 0.05.

## 3. Results

### 3.1. Sociodemographic, Clinical and Sexual Factors of The Participants

Out of 220 study participants approached, 219 completed the survey, giving a response rate of 99%. A total of 218 sample were included in the analysis. One outlier with a duration of diagnosis of 108 months (9 years) was dropped from the study analysis as it did not appropriately reflect the target population being studied. The mean (SD) age of the participants was 22.28 (SD: 1.77). The majority of the study participants were male (97.7%), Malay (80.7%), single (98.6%), obtained a formal education in a university (74.3%) and was working either full time or part time (66.5%). In terms of clinical factors, slightly more than half (56.0%) were follow-up patient with mean (SD) duration of follow-up of 16.48 months (SD: 16.68). Meanwhile, 52.3% of the participants were currently diagnosed with either HIV or STDs, in which 55.3% of those diagnosis were made within the previous year. Regarding the sexual factors, approximately two-thirds (64.2%) of the participants were sexually active within the past three months, had more than one sexual partner (66.5%) and had first sexual intercourse after 18 years old (63.8%). Apart from that, 41.7% of the participants were not aware of their sexual partner HIV status. A majority of the participants practiced anal receptive (34.3%) followed by oral sex (31.8%), anal insertive (24.8%) and vagina (9.1%) sexual practice. Among the study participants, two-thirds (67.0%) were gay/lesbian and the remaining one-third was heterosexual (22.0%) and bisexual (11.0%). The study participants’ sociodemographic, clinical and sexual factors are shown in Table 1.

### 3.2. Condom Usage

Of all the study participants, 61% (95% CI: 0.54, 0.68) reported good condom usage by using a condom during the last sexual intercourse and have the intention to use a condom in the next sexual intercourse. Meanwhile, another 39% reported to have poor condom usage. Among the participants who had poor condom usage, most of them did not use a condom in their last sexual intercourse but have the intention to use a condom in their next sexual intercourse (33.5%). Table 2 shows the prevalence of good and poor condom usage.

### 3.3. Condom Use Self-Efficacy

The average mean total score of condom use self-efficacy was 3.07. The assertiveness subscale had the highest score with average mean score of 3.42, while the intoxicant subscale had the lowest score with average mean score of 2.24. Table 3 demonstrates the scores for condom use self-efficacy and its subscale.

### 3.4. Factors Associated with Good Condom Usage

Table 4 shows the result from the simple logistic regression (SLR). Eleven variables were found to have a *p*-value of <0.05. These were age, type of visit, duration of follow up in HIV/STDs clinic, currently diagnosed with HIV/STDs, duration diagnosis of HIV/STDs, sexual orientation, sexual practice of anal insertive, condom use during first sexual intercourse and 3 subscale scores of condom use self-efficacy (mechanic subscale score, perceived barrier subscale score and assertiveness subscale score). These factors were included in the multiple logistic regression (MLogR).

Table 5 shows the factors associated with good condom usage using multiple logistic regression analysis. One outlier with a duration of diagnosis of 108 months (9 years) was dropped from the study analysis as it did not appropriately reflect the target population being studied. The model fitness was evaluated using Hosmer–Lemeshow goodness-of-fit test, which was not significant (*p* = 0.18). This demonstrates that the model fits the data well. The model explains 36.9% (Nagelkerke R Square) of the variance of the good condom usage. The classification table shows a specificity of 64.9%, indicating that the model could correctly classify 64.9% of the participants who are poor condom usage. The model’s sensitivity is 89.6%, indicating that the model could accurately classify 89.6% of participants who practice good condom usage. This model is able to discriminate 80.8% (ROC = 0.808, 95% CI: 0.71, 0.90, *p* < 0.05) of participants who are either good or poor condom usage.

The multiple logistic models identified three significant factors of good condom usage. Participants who used a condom during their first sexual intercourse had almost six times the odds to be a good condom user compared to those who did not [OR = 5.81 (95% CI: 1.97, 17.14, *p*-value 0.001)]. Participants diagnosed with HIV/STDs for a longer duration (more than 12 months) were 6.4 times more likely to be a good condom user compared to shorter duration of diagnosis (twelve or less than 12 months) [OR = 6.40, 95% CI: 2.30, 17.86, *p*-value < 0.001)]. Finally, the increase in score of assertiveness subscale by 1 increases the odds of good condom usage by 1.19 times, while controlling for confounding factors [OR = 1.19, 95% CI: 1.03, 1.36, *p*-value 0.017)].

## 4. Discussion

This study determines the prevalence of condom usage and explores the factors associated with condom usage in youth population attending HIV/STDs clinic at government primary care clinic in Selangor state of Malaysia. The prevalence of condom usage among the study population is 61.0% (95% CI: 0.54, 0.68) which is higher compared to the national data. The National Health and Morbidity Survey (NHMS), Malaysia has reported lower prevalence of condom usage (12.7%) among high school students. In Selangor state of Malaysia, the prevalence of sexually active youth is the highest (7.1%) among the states in Malaysia but only 11.7% of them reported condom usage during the last sexual intercourse [2]. The higher prevalence of condom usage among our study findings can be explained by the selection of study sites and population. Our study sampled youth from selected primary care clinics that offer expanded and specialised services for HIV/STDs. In addition to that, since youth in our study attended the HIV/STDs clinic, they would be motivated to look after their sexual health and practice safe sex through condom usage. Studies have proven that clinic-based counselling is associated with the increase in condom usage [34]. Comparing it with developed countries, the studies conducted in developed Western countries such as Great Britain, Denmark, Finland, France, Spain and Switzerland reported that the prevalence of condom usage among youth is over 40% [12,35] which is comparable to our findings. These findings might be contributed to condom initiative programs that they implement among youth [36,37].

There are three important clinical and behavioural factors associated with condom usage that have been identified in this study and might be useful in developing an effective HIV/STDs prevention program among the youth. In this study, we found that condom use during the first sexual intercourse is associated with condom usage. This finding is similar with previous studies conducted among the youth in Canada, Eastern China and South Africa that demonstrate condom use during the first sexual intercourse is one of the key determinants of subsequent condom usage [38,39,40]. Condom use during first sexual intercourse demonstrates condom consideration and consciously preparing for safe sex before becoming sexually active. Individuals who use condom during their first sexual intercourse are shown to exhibit consideration towards condom in preparing themselves for a safe sex before becoming sexually active [40]. Sex education may have an impact on such responsible choice and preparation during their first sexual intercourse [41]. Findings in this study suggest that early sexual education for youth before they become sexually active enables them to make informed choices about their future sexual safety. This ultimately may have a major impact on the prevention of HIV/STDs among youth in Malaysia.

In terms of clinical factors, duration diagnosis of HIV/STDs is found to be associated with condom usage. In this study, youth with longer duration diagnosis of HIV/STDs (more than 12 months) are more likely to use condom. Consistent with previous study, the longer diagnosis duration (more than 5 years) is associated with a higher likelihood of condom usage [42]. This finding might be due to the continuous health education during follow-up, which makes them aware on the risk of transmission of diseases to their partner and potentially use condom. In addition, the longer the youth has been receiving education, the more knowledge they would gain, and the better adherence they would have in applying that knowledge, particularly on condom usage [12]. However, another study found no significant association between duration of diagnosis of HIV/STDs with condom usage [43]. They may have believed that a lengthier diagnosis and course of treatment would lower their risk of HIV/STD transmission, but the finding of this study shows that this is not the case.

One important finding of this study is that a higher assertiveness score is found to be positively related to condom usage. Previous studies demonstrated mixed findings. Addoh et al. found no statistically significant association between the assertiveness self-efficacy scale and condom usage [44]. However, several studies reported significant association between assertiveness and condom usage [45,46]. The study among university students in Eastern China found that assertiveness in using condom before sex is a potent predictor of condom usage [45]. Another study by Uribe-Alvarado et al. showed that sexual assertiveness is strongly related to condom usage among young Colombian university students [46]. A potential reason for the high assertiveness score of this study could be attributed to the study sampling site. Youth who participated in this study may have received ongoing behavioural counselling while visiting the HIV/STDs clinic. In addition, the study’s population is at-risk youth who are sexually experienced and they might have developed the skill of assertiveness during their sexual encounter. According to the study, sexual inexperience is linked to low assertiveness [47].

Assertiveness is the key determinant in communicating the desire for condom use through actively disagreeing, expressing positive or negative personal rights and feelings, and standing up for one’s self without attacking another [48]. This ability to manifest openly and respectfully to the partner the needs, wishes and feelings about the condom usage without going against the right of the other person will determine how that person feels, thinks, and self-motivates to engage in condom usage in the future [46]. This could be the reason of assertiveness being one of significant factors in our study. There are several factors influencing sexual assertiveness including demographic variables (e.g., age, education, gender), sexual experiences (e.g., type of partnership, sexual victimization), psychosexual issues (e.g., sexual functioning, body self-esteem, emotion regulation, resourcefulness) and cultural factors (e.g., sexual scripts, gender stereotypes) [49]. These findings provided useful information that can be incorporated into the health education program of sexual behaviour among high-risk youth. To enhance condom usage among high-risk youth, the education or counselling must focus on developing skills in assertiveness as an essential component.

The rising prevalence of HIV/STDs among youth in Malaysia is worrying and the high prevalence of gay/lesbian youth in our study needs to be addressed [5]. Malaysia is known for its conservative sexual attitude and the sexual behaviour of Malaysians is highly influenced by the dominant Malay culture and Muslim religious belief [13]. Thus, issues on sexuality and condom use are rarely discussed. Therefore, any strategy targeted towards sexual education and condom use is best to be conducted judiciously in a culturally sensitive and acceptable manner [50]. Such programs should not be limited to condom usage per se, but should also address psychosocial factors such as assertiveness adequately and effectively encourage safer behaviour.

### 4.1. Strengths, Limitations and Implications for Future Research

As far as the researchers are concerned, this study is the first local study to be conducted among the youth attending HIV/STDs clinics in the primary care setting in Malaysia that studied the subject related to condom usage among youth. Therefore, this study fills an important gap in the literature and provides pertinent data for sexual-health promotion among youth in HIV/STDs clinic in Selangor, Malaysia.

However, this study has several limitations. First, the cross-sectional design precluded conclusions to be reached about causal relationships. This study would only show the relationship between the predictive variables and good condom usage. The results should be taken in considering the fact that this study’s design would only reveal the relationships between variables, not their causality.

Second, the use of convenience sampling of youth may introduce selection bias. A convenience sampling result would also not be representative of the population since there is a possibility of under- or over-representation of the population. However, measures were taken to reduce the sampling bias by including only youth, excluding adults and ensuring every youth patient listed on the HIV/STDs clinic registry during the data collection days was approached for participation.

Furthermore, this study is also confined within the urban population; therefore, caution should be taken when interpreting the findings and generalizing them. In addition, as this study relied on self-reporting by the youth, their responses could possibly reflect a socially desirable response with regard to reported condom use and self-efficacy among young adults.

In view of time limitation, we cannot exclude the possibility of unmeasured confounding factors that might affect the study findings, as many other factors can contribute towards good condom usage in youth attending HIV/STDs clinics, such as knowledge on HIV/STDs and condom, condom use attitudes and perceived risk and susceptibility to HIV/STDs. Thus, the understanding of other culturally sensitive factors that are relevant to the condom usage among youth population within the local context is an area of opportunity for further research.

### 4.2. Implications for Clinical Practice

The findings of this study have several implications for clinical practice. The current early sexual education at school should be emphasised as to empower youth to use condoms before they become sexually active. Comprehensive sexual education made as a standalone subject, instead of a component of physical education, or a religious or science subject, might assist in getting the right information about sex from a qualified source as opposed to the internet or from their peers, and is a key factor in reducing HIV/STDs.

The real challenge being faced is not convincing the youth that condoms work but rather finding the means of establishing a belief that they possess the power and skills to enact the solution. Education on the assertiveness of condom use to the youth with high-risk sexual behaviour should be emphasised to enhance their skills, such as negotiation of condom usage with sexual partners and to be assertive when the partner refuses to do so. Campaigns targeted on high-risk group and motivational counselling at clinics are cheap interventions that could be implemented. This may include individual counselling, focused group activity, or health promotion to increase condom use self-efficacy among the targeted group. It can be beneficial in preventing HIV/STDs transmission among youth in the future.

## 5. Conclusions

In conclusion, youth in this study reported a high prevalence of good condom usage. This study identified condom use during first sexual intercourse, longer duration of HIV/STDs diagnosis, and assertiveness of condom use self-efficacy are positively affecting good condom usage among the youth attending HIV/STDs clinics in Malaysia. The assertiveness in condom usage, among other factors in condom use self-efficacy, is an important factor to facilitate good condom usage among the youth. These findings provided useful information that can be incorporated into strategies to improve condom usage among the youth especially those who are in the high-risk group.

## Figures and Tables

**Table 1:** Sociodemographic, clinical and sexual factors of the participants, *n* = 218.

Variables	Frequency, *n* (%)	Mean (SD)
**Sociodemographic factors**		
**Biological sex**		
Male	213 (97.7)	
Female	5 (2.3)	
**Age (years)**		22.28 (1.77)
**Race**		
Malay	176 (80.7)	
Chinese	13 (6.0)	
Indian	9 (4.1)	
Others	20 (9.2)	
**Marital status**		
Single	215 (98.6)	
Married	3 (1.4)	
**Highest education level**		
No formal education/Primaryschool/Secondary school	41 (18.8)	
Pre-university (STPM/Matriculation)	15 (6.9)	
University (Diploma/Degree/Masters/PhD)	162 (74.3)	
**Religion**		
Muslim	184 (84.4)	
Christian	9 (4.1)	
Buddhist	10 (4.6)	
Hindu	9 (4.1)	
Others	6 (2.8)	
**Occupation**		
Unemployed	10 (4.6)	
Student	63 (28.9)	
Full time/part time worker	145 (66.5)	
**Clinical Factors**		
**Type of visits**		
New visit	96 (44.0)	
Follow-up visit	122 (56.0)	
**Duration follow up in HIV/STDs clinic (months)**		16.48 (16.68)
**Currently diagnosed with HIV/STDs**		
Yes	114 (52.3)	
No	104 (47.7)	
* Duration diagnosis of HIV/STDs		
≤12 months	63 (55.3)	
>12 months	51 (44.7)	
* Currently receiving treatment for HIV/STDs		
Yes	110 (96.5)	
No	4 (3.5)	
**Past history of STDs**		
Yes	78 (35.8)	
No	140 (64.2)	
**Alcohol consumption**		
Yes	78 (35.8)	
No	140 (64.2)	
**Illicit drug consumption**		
Yes	28 (12.8)	
No	190 (87.2)	
**Smoking status**		
Yes	59 (27.1)	
No	159 (72.9)	
**Sexual Factors**		
**Sexually active past 3 months**		
Yes	140 (64.2)	
No	78 (35.8)	
**Sexual orientation**		
Heterosexual	24 (11.0)	
Gay/lesbian	146 (67.0)	
Bisexual	48 (22.0)	
**† Sexual practice**		
Vaginal sex	40 (9.1)	
Oral sex	140 (31.8)	
Anal Insertive	109 (24.8)	
Anal Receptive	151 (34.3)	
**Number of sexual partner/s**		
One	73 (33.5)	
More than one	145 (66.5)	
**Age of 1st sexual intercourse (years)**		
Less than 18	79 (36.2)	
18 and above	139 (63.8)	
**Condom use during 1st sexual intercourse**		
Yes	91 (41.7)	
No	127 (58.3)	
**Aware of sexual partner HIV status**		
Yes	91 (41.7)	
No	127 (58.3)	

* Included only participants who are currently diagnosed with HIV/STDs, *n* = 114. † Multiple responses, *n* = 440.

**Table 2:** Prevalence of good and poor condom usage, *n* = 218.

Variables	Frequency, *n* (%)
No condom usage (Score 0)	10 (4.6)
Used condom at last sexual intercourse but do not intend to use condom in next sexual intercourse (Score 1)	2 (0.9)
Did not use condom at last sexual intercourse but intend to use condom in next sexual intercourse (Score 1)	73 (33.5)
Used condom at last sexual intercourse and intend to use condom in next sexual intercourse (Score 2)	133 (61.0)
**Condom usage scores**	
Poor condom usage (Score 0–1)	85 (39.0)
Good condom usage (Score 2)	133 (61.0)

**Table 3:** The mean scores for condom use self-efficacy and its subscale.

Variables	Mean (SD)	Average Mean Score Per Item (SD)	Minimum	Maximum
**Total CUSES score (0–108)**	82.93 (14.29)	3.07 (0.53)	38.00	108.00
Mechanic subscale score (0–40)	32.53 (5.69)	3.25 (0.57)	11.00	40.00
Perceived barrier subscale score (0–28)	19.71 (6.91)	2.81 (0.99)	1.00	28.00
Assertiveness subscale score (0–28)	23.96 (3.68)	3.42 (0.53)	7.00	28.00
Intoxicant subscale score (0–12)	6.73 (2.62)	2.24 (0.87)	0.00	12.00

**Table 4:** Simple logistic regression on factors associated with good condom usage.

Variables	Beta	Standard Error (df)	OR (95% CI)	*p*-Value
**Biological sex**				
Male	1.88	1.13	6.52 (0.72, 59.34)	0.096
Female			1	
**Age (years)**	0.16	0.08	1.18 (1.01, 1.37)	0.040
**Race**				
Malay	0.15	0.48	1.17 (0.45, 3.00)	0.749
Chinese	−0.56	0.72	0.57 (0.14, 2.34)	0.437
Indian	−1.10	0.84	0.33 (0.06, 1.74)	0.192
Others			1	
**Marital status**				
Single	-	-	-	
Married	-	-	-	0.283 †
**Highest education level**				
No formal education/Primary school/Secondary school			1	
Pre-university (STPM/Matriculation)	0.18	0.61	1.200 (0.37, 3.92)	0.763
University (Diploma/Degree/Masters/PhD)	0.66	0.35	1.93 (0.97, 3.86)	0.062
**Religion**				
Muslim	1.25	0.88	3.49 (0.62, 19.58)	0.155
Christian	1.39	1.12	4.00 (0.45, 35.79)	0.215
Buddhist	0.69	1.07	2.00 (0.24, 16.36)	0.518
Hindu	0.00	1.12	1.00 (0.11, 8.95)	1.000
Others			1	
**Occupation**				
Unemployed			1	
Student	−0.05	0.69	0.95 (0.24, 3.70)	0.940
Full time/part time worker	0.09	0.67	1.091 (0.30, 4.04)	0.896
**Type of visit**				
New visit			1	
Follow up visit	0.59	0.28	1.81 (1.04, 3.12)	0.035
**Duration of follow up in HIV/STDs clinic (months)**	0.04	0.02	1.04 (1.01, 1.07)	0.012
**Currently diagnosed with HIV/STDs**				
Yes	0.58	0.28	1.78 (1.03, 3.09)	0.039
No			1	
* Duration diagnosis of HIV/STDs				
≤12 months			1	
>12 months	1.74	0.48	5.71 (2.24, 14.61)	<0.001
* Currently receiving treatment for HIV/STDs				
Yes	0.76	1.02	2.14 (0.29, 15.84)	0.455
No			1	
**Past history of STDs**				
Yes	0.20	0.29	1.23 (0.69, 2.17)	0.485
No			1	
**Alcohol consumption**				
Yes			1	
No	0.55	0.29	1.73 (0.98, 3.04)	0.057
**Illicit drug consumption**				
Yes			1	
No	0.68	0.41	1.98 (0.90, 4.40)	0.094
**Smoking status**				
Yes			1	
No	0.58	0.31	1.78 (0.97, 3.26)	0.062
**Sexually active past 3 months**				
Yes	−0.38	0.30	0.69 (0.39, 1.22)	0.202
No			1	
**Sexual orientation**				
Heterosexual			1	
Gay/lesbian	1.20	0.47	3.31 (1.33, 8.24)	0.010
Bisexual	1.58	0.54	4.86 (1.70, 13.91)	0.003
**Sexual practice**				
**Vaginal sex**				
Yes			1	
No	0.05	0.36	1.05 (0.52, 2.12)	0.885
**Oral sex**				
Yes	0.30	0.29	1.35 (0.77, 2.37)	0.299
No			1	
**Anal insertive**				
Yes	0.66	0.28	1.94 (1.12, 3.37)	0.019
No			1	
**Anal receptive**				
Yes	0.44	0.30	1.55 (0.86, 2.77)	0.143
No			1	
**Number of sexual partner/s**				
One			1	
More than one	−0.22	0.30	0.81 (0.45, 1.44)	0.469
**Age of 1st sexual intercourse (years)**				
Less than 18			1	
18 and above	0.18	0.29	1.20 (0.68, 2.11)	0.526
**Condom use during 1st sexual intercourse**				
Yes	1.22	0.31	3.39 (1.86, 6.16)	<0.001
No			1	
**Aware of sexual partner HIV status**				
Yes	0.28	0.28	1.32 (0.76, 2.30)	0.327
No			1	
**Mechanic subscale score (Score 0–40)**	0.10	0.03	1.10 (1.05, 1.16)	<0.001
**Perceived barrier subscale score (Score 0–28)**	0.05	0.02	1.05 (1.01, 1.09)	0.020
**Assertiveness subscale score (Score 0–28)**	0.15	0.04	1.16 (1.07, 1.26)	<0.001
**Intoxicant subscale score (Score 0–12)**	0.05	0.05	1.05 (0.95, 1.17)	0.329

1 = Reference group Emboldened: Statistical significance at *p* < 0.05. * Included only participants who are currently diagnosed with HIV/STDs, *n* = 114. † Analyse using χ^2^ test.

**Table 5:** Multiple logistic regression on factors associated with good condom usage.

Variables	Adjusted Beta	Adjusted Standard Error (df)	Adjusted OR (95% CI)	*p* Value
**Condom use during 1st sexual in-tercourse**				
Yes	1.76	0.55 (1)	5.81 (1.97, 17.14)	0.001
No			1	
**Duration diagnosis of HIV/STDs (months)**				
≤12 months			1	
>12 months	1.86	0.52 (1)	6.40 (2.30, 17.86)	<0.001
**Assertiveness subscale score**	0.17	0.07 (1)	1.19 (1.03, 1.36)	0.017

The model fits well (χ^2^ = 11.42, df = 3, N = 218, *p* < 0.05) Model assumptions were met, no significant interaction and multicollinearity.

## Data Availability

Data are kept at the Department of Primary Care Medicine, Universiti Teknologi MARA, in Selangor, Malaysia. Data may be shared upon reasonable request and are subjected to the data protection laws and regulations.
